# Identification of Candidate Genes for Twinning Births in Dezhou Donkeys by Detecting Signatures of Selection in Genomic Data

**DOI:** 10.3390/genes13101902

**Published:** 2022-10-19

**Authors:** Taifeng Xie, Shuer Zhang, Wei Shen, Guoliang Zhang, Rong Guo, Wei Zhang, Yanhang Cao, Qingjie Pan, Fengxin Liu, Yujiang Sun, Shuqin Liu

**Affiliations:** 1 College of Animal Science and Technology, Qingdao Agricultural University, Qingdao 266109, China; 2 Shandong Animal Husbandry General Station, Jinan 250022, China; 3 College of Life Sciences, Qingdao Agricultural University, Qingdao 266109, China; 4 Shandong Equine Animal Genetic Resources Gene Bank, Qingdao 266109, China; 5 Dongying Modern Animal Husbandry Development Service Center, Dongying 257091, China; 6 Shandong Dezhou School, Dezhou, 251500, China; 7 Vocational College of Dongying, Dongying 257091, China

**Keywords:** twinning trait, Dezhou donkey, whole genome resequencing, reproductive hormone

## Abstract

Twinning trait in donkeys is an important manifestation of high fecundity, but few reports are available elucidating its genetic mechanism. To explore the genetic mechanism underlying the twin colt trait in Dezhou donkeys, DNA from 21 female Dezhou donkeys that had birthed single or twin colts were collected for whole-genome resequencing. F_ST_, θπ and Tajima’s D were used to detect the selective sweeps between single and twin colt fecundity in the Dezhou donkey groups. Another set of 20 female Dezhou donkeys with single or multiple follicles during estrus were selected to compare concentrations of reproductive hormone including follicle-stimulating hormone (FSH), luteinizing hormone (LH), estradiol (E_2_) and progesterone (P_4_). Four candidate genes including *ENO2*, *PTPN11*, *SOD2* and *CD44* were identified in the present study. The *CD44* gene had the highest F_ST_ value, and *ENO2*, *PTPN11* and *SOD2* were screened by two joint analyses (F_ST_ and θπ, θπ and Tajima’s D). There was no significant difference in the LH, FSH and P_4_ levels between the two groups (*p* > 0.05); however, the serum E_2_ content in the multi-follicle group was significantly higher than that in the single-follicle group (*p* < 0.05). The identified candidate genes may provide new insights into the genetic mechanism of donkey prolificacy and may be useful targets for further research on high reproductive efficiency.

## 1. Introduction

Litter size is a complex reproductive trait and a research hotspot that has attracted extensive academic attention. An increase in litter size impacts livestock husbandry by increasing production. Genes such as *FecB*, *FecX*, *GDF9*, *FecL*, *LEPR*, *CPR54*, *BMPR-IB*, *KISS1* and *FSHB* have been identified as major factors influencing the fecundity of domestic animals [1,2,3,4,5]. In their natural reproductive state, multiple births in livestock are mainly related to the number of ovulations, and the effect of hormones on ovulation is crucial. Correlation exists between follicular development and serum level of reproductive hormones, and litter size is highly correlated with ovulation number; the greater the ovulation number, the higher the number of offspring [6,7,8]. The ovulation rate in sheep can be increased by increasing exogenous FSH [9]; the higher the FSH/LH ratio, the greater the litter size [10]. Ewes with high fertility have higher concentrations of FSH and E_2_ than do low-fertility ewes [11]. In addition, the use of P_4_ + eCG for synchronous estrus could improve the overall pregnancy and lambing rates of Ghezel ewes in practice [12].

The Dezhou donkey is one of five most popular and widely distributed domestic donkey breeds in China, known for their excellent meat and skin production and also being used for labor. The occurrence of twin births is common in Dezhou donkey production. In the past, multiple births or twinning in equids were regarded as unfavorable as it led to the death or shortened lifespan for the offspring. However, for now, the survival rate of twin donkeys has been increased significantly with improvements in nutritional quality and living conditions. Hence, studies on the mechanisms and factors affecting donkey prolificacy are useful for increasing donkey production and profitability.

Compared with other domestic animals, donkeys have a longer pregnancy (average 365 days), which greatly limits donkey breeding. Under natural physiological conditions, the ovaries of female donkeys mostly develop a single follicle during each estrus stage. The rate of development of multiple follicles is only 5.3–38.1%, and the probability of multiple follicular development in large donkeys is 61% higher than that in medium and small donkeys [13]. The mean prevalence of multiple births per hundred births in the Andalusian donkey population is 9.73% [14]. Whether a relationship exists between reproductive hormones and the twinning trait in donkeys is yet unconfirmed.

In this study, whole genome resequencing (WGS) was used for performing comparative population genomics to identify candidate genes responsible for the twinning trait in Dezhou donkeys. Serum reproductive hormone concentrations were measured using enzyme-linked immunosorbent assay (ELISA) in single and multiple follicles development events during estrus.

## 2. Materials and Methods

### 2.1. Sample Collection

A total of 21 female donkey blood samples were collected for whole genome resequencing, which were characterized by single birth (*n* = 10) and twinning birth (*n* = 11). Another 20 female donkey blood samples were collected for the determination of serum reproductive hormone during estrus, which were characterized by single-follicle (*n* = 10) and multi-follicles (*n* = 10). Blood samples from these 41 female donkeys were collected from the Dezhou Donkey National Conservation Farm. Genomic DNA was isolated from 21 blood samples using the centrifugal column method and preserved at −20 °C. The amount of DNA was determined by measuring the A260/A280 value using NanoDrop 2000, followed by further evaluation using 1% agarose gel electrophoresis. Serum was isolated from 20 blood samples and stored at −80 °C. All samples were obtained in compliance with the principles approved by the Animal Care and Use Committee of Qingdao Agricultural University.

### 2.2. Whole Genome Resequencing and Analysis

High quality genomic DNA obtained from 10 donkeys with single birth (single colt group) and 11 donkeys with twinning birth (twin colt group) was fragmented. After a process in which it was end-repaired, A-tailed, ligated to paired-end adaptors and underwent PCR amplification with 400 bp inserts, the constructed DNA library was sequenced using Illumina Hiseq system for 150 bp paired-end reads at Shanghai Majorbio Bio-pharm Technology Co., Ltd. (Shanghai, China). After the low-quality reads were filtered, a series of steps were carried out, including (1) removing the adapter sequence in reads; (2) cutting off the bases whose 5′-end and 3′-end sequencing quality value was lower than 20 or identified as N; (3) cutting off the bases in the window whose average quality value was less than 20; (4) filtering out reads with undetected bases exceeding 10% of the total sequence length; (5) cutting off more than 40% of the reads whose Q ≤ 15; (6) removing adapter and remove reads with a length less than 30 bp after quality trimming. The high-quality data were aligned to the donkey reference genome (https://www.ncbi.nlm.nih.gov/data-hub/genome/GCF_016077325.2/, accessed on 14 October 2021) using the software Burrows Wheeler Aligner (BWA) under default mapping parameters [15]. Molecular markers were called and filtered using GATK 3.8.0 [16]. Variation function annotation was completed according to SnpEff software and the gene prediction information of the donkey reference genome [17]. The SNP functional annotation information results were subjected to further quality control (with a set deletion rate of 30%) to obtain high-quality SNP data for subsequent population genetic diversity and selection signal analysis.

Principal component analysis (PCA) was carried out in the GCTA software program EIGENSTRAT based on the GREML analysis [18]. The decay of linkage disequilibrium (LD) measured by the correlation coefficient (r^2^), between adjacent SNPs across the complete genome was calculated using VCFtools [19]. Based on the high-quality SNPs, a neighbor-joining phylogenetic tree (NJ tree) was generated by FastTree [20].

To detect regions with significant signatures of selective sweep, three methods (F_ST_, θπ and Tajima’s D) were used to identify candidate twinning-trait regions using VCFtools. Sliding window size of 100 kb and a step size of 10 kb was performed across the genome, the top 5% of F_ST_ and the smallest 5% of θπ were selected, the top 5% and the smallest 5% of Tajima’s D were selected, and then joint analyses (F_ST_ and θπ, θπ and Tajima’s D) were performed to extract the corresponding candidate regions and variation sites information.

### 2.3. Gene Set Enrichment and Pathway Analysis

To identify the possible pathways for the strong selective regions involved in the regions with top values (joint analysis), Kyoto Encyclopedia of Genes and Genomes (KEGG) pathway analysis was performed using the ‘kobas.cbi.pku.edu.cn/annoiden.php’(accessed on 9 September 2022) enrichment analysis tool to uncover candidate gene biological functions.

### 2.4. Hormone Level Measurement and Analysis

Serum samples were collected from 10 single-follicle and 10 multi-follicle donkeys (Figure S1). The serum E_2_, LH, FSH and P_4_ levels were determined using ELISA with commercially available kits (Nanjing Jiancheng Bioengineering Institute Co., Ltd., Nanjing, China) according to the manufacturer’s instructions. The optical density (OD) at 450 nm was measured using an ELISA microplate reader (Tecan Infinite F50).

## 3. Results

### 3.1. Sequencing and Read Alignment

In this study, 21 DNA samples from Dezhou female donkeys were re-sequenced at a sequencing depth of 10×. A total of 563.31 G clean data were generated, with an average of 26.82 G clean data and an average coverage depth of 8.12× per sample. A total of 98.99–99.77% of the clean reads were mapped to the newly annotated donkey reference genome (GCF_016077325.2_ASM1607732v2) (Tables S1 and S2). A total of 8,995,441 SNPs and 1,671,190 InDels were identified. Through further quality control, a total of 131,407 SNPs were obtained from 21 individuals for subsequent population genetic diversity and selection signal analysis.

### 3.2. Phylogenetic Analysis and Linkage Disequilibrium Decay

**Figure 1:**
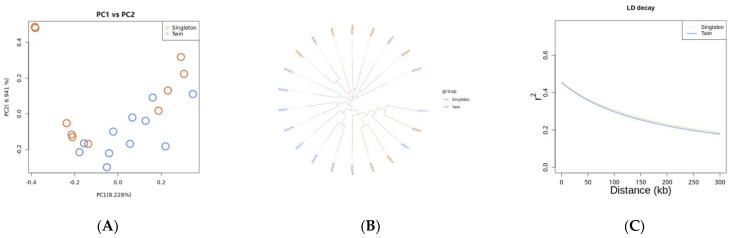
Population genetic diversity. (**A**) PCA plot of Dezhou donkey populations; (**B**) Neighbor-joining phylogenetic tree of Dezhou donkeys; (**C**) Linkage disequilibrium decay analysis.

Different classical analyses including NJ tree, PCA and LD were performed. The phylogenetic tree and PCA results showed that the twin colt group was relatively concentrated, whereas the single colt group was separated into two small branches but was nevertheless distinct from the twin colt group (Figure 1A,B). To investigate the effects of selection, the average LD level, measured based on r^2^, was estimated (Figure 1C). Within the population, LD decay of the two populations was nearly the same.

### 3.3. Positively Selected Genes of Dezhou Donkeys

**Figure 2:**
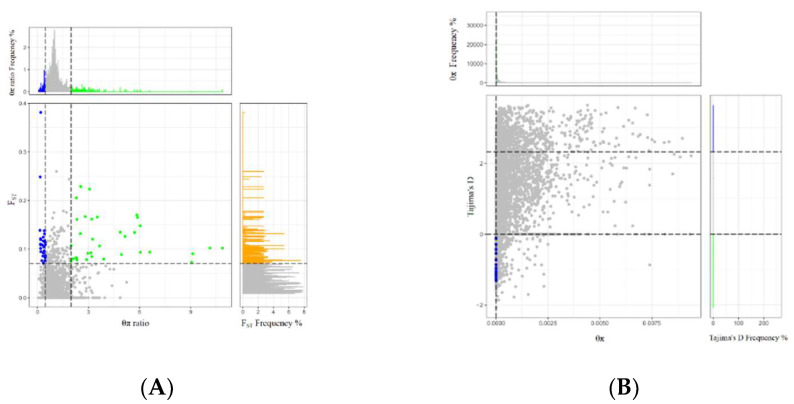
Intersection of the joint analysis used to identify selection regions. (**A**) Distribution of θπ ratios (θπ, single colt group /θπ, twin colt group) and F_ST_ values, which are calculated in 100 kb windows and sliding in 10 kb steps. Data above the horizontal dashed line (where F_ST_ is 0.0729208) were identified as selected regions for single colt group (blue points) and twin colt group (green points), respectively; (**B**) Distribution of θπ and Tajima’s D. Selection was taken by a 5% threshold, with blue and green dots as candidate loci.

To find variations in the genomes of donkeys in the single colt group and twin colts group, two joint methods (F_ST_ and θπ, θπ and Tajima’s D) were used to localize the selection signal with a 5% threshold for selection. The distribution of corresponding values for the selected regions is shown in Figure 2. At the 5% level, 243 candidate regions and 113 genes were obtained through the joint analysis of F_ST_ and θπ (Figure 2A), whereas 919 candidate regions and 341 genes were obtained through joint analysis of θπ and Tajima’s D (Figure 2B).

**Figure 3:**
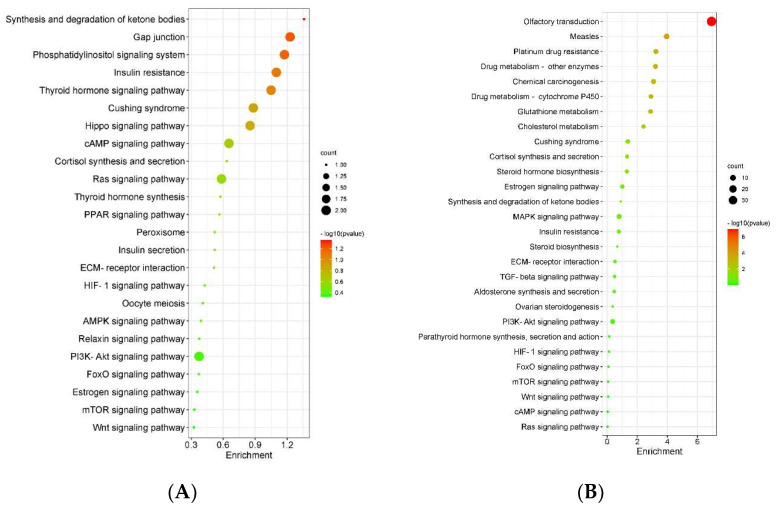
The KEGG analysis for candidate genes related to reproductive traits and significant pathways in the twin colt group. (**A**) F_ST_ and θπ; (**B**) θπ and Tajima’s D.

The KEGG pathway enrichment analysis by F_ST_ and θπ combined analysis revealed that all genes were enriched in 24 pathways related to reproductive traits, including 13 genes related to reproduction (Figure 3A and Table 1). And gene *CD44* of them had the highest F_ST_ value, was enriched in the ECM-receptor interaction pathway. Meanwhile, the KEGG pathway enrichment analysis by the joint analysis of θπ and Tajima’s D revealed 22 pathways which were related to reproductive traits, and 30 of 341 genes were related to reproduction (Figure 3B and Table 2). Three common genes (*ENO2*, *PTPN11* and *SOD2*) found to be involved in reproductive traits when screened by both methods jointly. These overlapping genes were enriched in HIF-1 signaling, insulin resistance, Ras signaling, FoxO signaling and peroxisome pathways (Table 3).

### 3.4. Serum Reproductive Hormone Level Results

**Figure 4:**
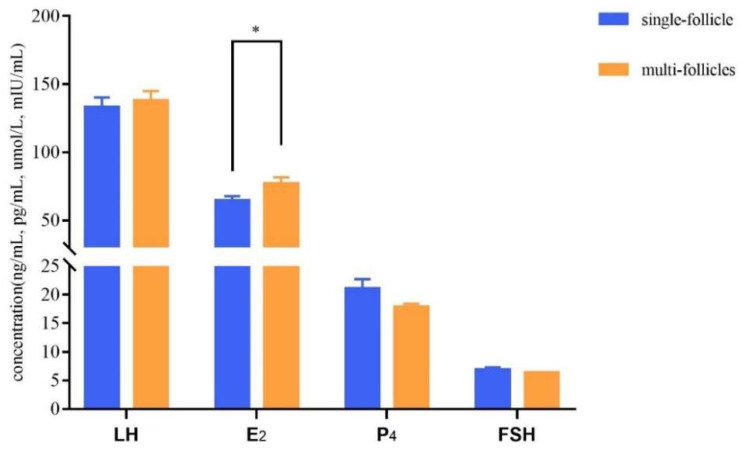
Concentrations of serum reproductive hormones. * *p* < 0.05.

With the aid of B-ultrasonic examinations, blood samples were collected from donkeys in the single-follicle and multi-follicle group during estrus. As shown in Figure 4, the serum E_2_ level in the multi-follicle group was significantly higher than that in the single follicle group (*p* < 0.05). However, no significant difference was detected in the contents of LH, FSH and P_4_ between the two groups (*p* > 0.05). The FSH and P_4_ levels in the single-follicle group were slightly higher than those in the multi-follicle group.

## 4. Discussion

In this study, a method for detecting selective sweeps was used to identify the candidate genes affecting litter size in donkeys. *ENO2*, *PTPN11* and *SOD2* were identified through two conjoint analyses (F_ST_ and θπ, θπ and Tajima’s D) and *CD44* had the highest F_ST_ value identified by the joint analysis of F_ST_ and θπ.

ENO plays an important role in animal growth. In vertebrates, ENO has three subunits, α, β and γ, and the homodimer ENO2, which is composed of γ subunits, exists in neurons and neuroendocrine tissues. LH and FSH upregulated *ENO2* expression in oocytes and theca cells, activating glycolysis in these cells [21], suggesting that LH and FSH may be involved in changes of *ENO2* mRNA expression during the estrous cycle, and activate the ovarian function by stimulating the glycolysis in oocytes and theca cells. Meanwhile, an increased E_2_ level has a negative feedback effect on the FSH level [22]. In this study, the content of serum E_2_ in the multi-follicle group was significantly higher than that in the single-follicle group, and the FSH level of the single follicle group was slightly higher than that in the multi-follicle group. These results are consistent with those of the earlier studies. 

*PTPN11* encodes the protein SHP2. SHP2 binds to RAS and dephosphorylates to increase RAS-RAF binding and activate downstream RAS/ERK/MAPK proliferative signaling, suggesting that SHP2 was a direct activator of RAS [23]. The response of SHP2 to growth factors, hormones and cytokines can be attributed to its dramatic effect on the activation of the Ras/MAPK cascade, and SHP2 catalytic activity is directly involved in the activation of many protein kinases expressed in oocytes and cumulus cells that control oocyte maturation and embryo development. SHP2 is necessary for the activation of the PI3K/Akt signaling pathway, which is abundantly expressed in oocytes and plays an important role in oocyte maturation and development [24,25]. This study showed that *PTPN11* was also enriched in the Ras signaling pathway, but how SHP2 precisely regulated the Ras/ERK/MAPK pathway is unclear and needs further research.

SOD2 primarily protects macromolecules, proteins, lipids and DNA from oxidative damage. SOD2 deficiency results in ROS-induced oxidative stress, which can attenuate the production of P_4_ and E_2_ in ovarian granulosa cells, and ROS/oxidative stress plays a key role in the negative regulation of steroidogenesis [26]. The results of this study showed that the E_2_ concentration in the twin colt group was higher than that in the single colt group, whereas the P_4_ concentration in the single colt group was higher.

In addition, *CD44*, which is broadly distributed on the cell surface, had the highest F_ST_ value and was identified through the joint analysis of F_ST_ and θπ. During cumulus-oocyte complex (COCs) expansion, the accumulation of hyaluronic acids affects oocyte maturation mainly by interacting with CD44 to break gap junctions in COCs and control meiosis [27,28]. It has been proven that CD44 can influence reproductive performance by inducing the maturation of oocytes and can be used as a potential marker of oocyte quality for predicting oocyte development capacities [29,30].

Some additional genes apart from *ENO2*, *PTPN11*, *SOD2* and *CD44* were also enriched in reproductive-trait-related pathways. *CTNNB1* was enriched in four pathways: thyroid hormone signaling pathway, Wnt signaling pathway, Hippo signaling pathway and Cushing syndrome. The Wnt/CTNNB1 pathway is currently considered to be a considerable factor regulating ovarian steroidogenesis. *CTNNB1* is involved in estrogen biosynthesis and was demonstrated to have a potential role in Wnt signaling for ovarian steroidogenesis and follicular growth [31,32]. *CTNNB1* had been identified as a candidate gene associated with litter size in goats and Hu sheep [33,34]. *SP1* was screened for use in the present study. SP1 and SP3 can mediate P_4_ dependent induction in the endometrium, realizing the transformation of E_2_ to E_1_ in endometrial epithelium [35]. This study also showed that the E_2_ concentration in the multi-follicle group was higher than that in the single-follicle group, whereas the P_4_ concentration showed the opposite trend. Therefore, we hypothesized that down-regulation of *SP1* and *SP3* expression caused by the decrease in P_4_ concentration can lead to a higher E_2_ concentration, and perhaps SP1 plays a key role in this process; further studies are needed to validate these hypotheses. *YWHAH*, *CREB3L1*, *ITGA7*, *BRCA1*, *LOC106824701*, *LOC106828271* and *VWF* screened in the twin colt group were enriched in the PI3K/Akt signaling pathway. *AMHR2* and *MAP3K12* were confirmed to be screened from Laoshan dairy goats with high fecundity [36]. Some candidate genes (such as *YWHAH*, *CTNNB1*, *PLCE1*, *CREB3L1*, *SCARB1*, *SP1* and *TNFRSF1A*) appeared repeatedly in different signaling pathways; they may affect follicular development and ovulation, and their impact on the twin-colt trait needs to be further studied.

## 5. Conclusions

In the present study, whole genome resequencing was performed on donkeys with single and twin colt births to investigate genetic differences that affect the twinning trait. The results showed that *ENO2*, *PTPN11*, *SOD2* and *CD44* are candidate marker genes for follicular development and high fecundity.

## Figures and Tables

**Table 1:** Genes associated with reproductive traits in twin colt group revealed by F_ST_ and θπ.

Gene Symbol	Chr	POS1	POS2	KEGG Pathway Name
*CD44*	17	27,349,506	27,438,460	ECM-receptor interaction
*ENO2*	22	34,652,151	34,660,012	HIF-1 signaling pathway
*PTPN11*	8	44,348,167	44,434,134	Insulin resistance, Ras signaling pathway
*HMGCS1*	10	56,083,656	56,103,960	PPAR signaling pathway
*YWHAH*	8	29,177,234	29,188,784	Oocyte meiosis, PI3K-Akt signaling pathway, Hippo signaling pathway
*DEPDC5*	8	29,218,385	29,335,598	mTOR signaling pathway
*CTNNB1*	21	52,260,677	52,318,403	Thyroid hormone signaling pathway, Wnt signaling pathway, Hippo signaling pathway, Cushing syndrome
*SOD2*	28	18,109,120	18,118,565	FoxO signaling pathway, Peroxisome
*MTMR3*	8	30,884,359	31,026,820	Phosphatidylinositol signaling system
*LOC106847879*	7	13,897,153	13,901,144	Gap junction
*LOC106847891*	7	13,841,750	13,844,884	Gap junction
*PLCE1*	2	81,090,063	81,419,500	cAMP signaling pathway, Phosphatidylinositol signaling system, Ras signaling pathway, Thyroid hormone signaling pathway
*CREB3L1*	17	36,425,577	36,460,685	AMPK signaling pathway, cAMP signaling pathway, cortisol synthesis and secretion, estrogen signaling pathway, insulin resistance, relaxin signaling pathway

**Table 2:** Genes related to reproductive traits in twin colt group revealed by θπ and Tajima’s D.

Gene Symbol	Chr	POS1	POS2	KEGG Pathway Name
*BRCA1*	13	44,593,779	44,648,984	PI3K-Akt signaling pathway
*SP1*	22	15,684,299	15,725,420	Cortisol synthesis and secretion, Cushing syndrome, estrogen signaling pathway, parathyroid hormone synthesis, secretion and action, TGF-β signaling pathway
*AMHR2*	22	15,668,089	15,677,118	TGF-β signaling pathway
*MAP3K12*	22	15,612,257	15,628,331	MAPK signaling pathway
*PPP5C*	26	16,802,294	16,824,493	MAPK signaling pathway
*VAV1*	20	4,822,639	4,879,177	cAMP signaling pathway
*SOD2*	28	18,109,120	18,118,565	FoxO signaling pathway, Peroxisome
*LOC106848497*	2	7,602,719	7,607,118	Steroid hormone biosynthesis
*LOC106848499*	2	7,561,635	7,566,102	Steroid hormone biosynthesis
*LOC106822285*	8	60,378,708	60,381,460	Estrogen signaling pathway, MAPK signaling pathway
*CSNK2B*	8	60,245,559	60,250,603	Wnt signaling pathway
*LOC106825061*	8	60,365,325	60,369,097	Estrogen signaling pathway, MAPK signaling pathway
*AGT*	2	110,546,191	110,557,530	Aldosterone synthesis and secretion, cortisol synthesis and secretion, Cushing syndrome, insulin resistance
*KMT2A*	20	33,106,167	33,186,707	Cushing syndrome
*PTPN11*	8	44,348,167	44,434,134	Insulin resistance, Ras signaling pathway
*LOC106824701*	23	15,673,367	15,674,109	PI3K-Akt signaling pathway
*LOC106828271*	23	15,749,886	15,750,443	PI3K-Akt signaling pathway
*ENO2*	22	34,652,151	34,660,012	HIF-1 signaling pathway
*TNFRSF1A*	22	34,171,301	34,183,621	Insulin resistance, MAPK signaling pathway, mTOR signaling pathway
*VWF*	22	33,854,011	34,007,142	PI3K-Akt signaling pathway, ECM-receptor interaction
*LIPA*	2	85,429,933	85,468,230	Cholesterol metabolism, steroid biosynthesis,
*GPER1*	14	5,923,474	5,931,179	estrogen signaling pathway
*IL1A*	6	77,320,629	77,330,047	MAPK signaling pathway
*ITGA7*	22	12,568,065	12,588,863	PI3K-Akt signaling pathway, ECM-receptor interaction
*SCARB1*	8	48,886,461	48,957,378	Aldosterone synthesis and secretion, cholesterol metabolism, cortisol synthesis and secretion, Cushing syndrome, ovarian steroidogenesis
*LIPC*	2	177,653,935	177,795,387	Cholesterol metabolism
*ABCA1*	10	35,604,150	35,735,913	Cholesterol metabolism
*LOC106833490*	29	2,069,225	2,075,203	Cushing syndrome
*LOC106843311*	26	11,864,786	11,876,092	Steroid hormone biosynthesis
*ACAT1*	20	42,259,297	42,281,562	Synthesis and degradation of ketone bodies

**Table 3:** Overlapping genes associated with reproductive traits in twin colt group based on both joint analyses.

Gene Symbol	Chr	POS1	POS2	KEGG Pathway Name
*ENO2*	22	34,652,151	34,660,012	HIF-1 signaling pathway
*PTPN11*	8	44,348,167	44,434,134	Insulin resistance, Ras signaling pathway
*SOD2*	28	18,109,120	18,118,565	FoxO signaling pathway, Peroxisome

## Data Availability

The data that support those findings in this study are currently not publicly available. Please contact the corresponding author for further information.

## Supplementary Materials

The following supporting information can be downloaded at: https://www.mdpi.com/article/10.3390/genes13101902/s1. Figure S1: The development of follicle; Table S1: Summary of sequencing data; Table S2: Summary of mapping statistics.
