# Supplementary figures and images for: Identification of Candidate Genes for Twinning Births in Dezhou Donkeys by Detecting Signatures of Selection in Genomic Data
Source: Genes (Basel). 2022 Oct 19;13(10):1902. doi: 10.3390/genes13101902 (PMC9601833; doi:10.3390/genes13101902)

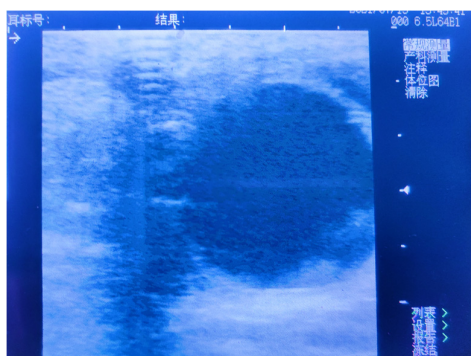

(a)

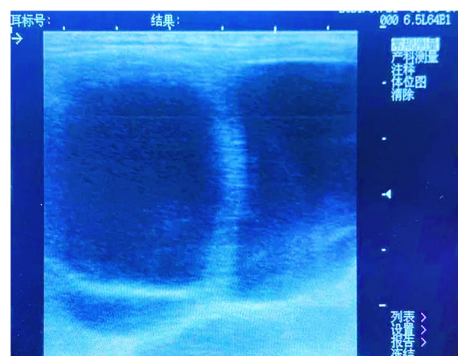

(b)

**Figure S1.** The development of follicle. (a) single follicle; (b) multiple follicles.

Supplement: Supplementary file 1 [file genes-13-01902-s001.zip › Supplementary files/Figure S1.pdf]
